# Development of Electronic Nose and Near Infrared Spectroscopy Analysis Techniques to Monitor the Critical Time in SSF Process of Feed Protein

**DOI:** 10.3390/s141019441

**Published:** 2014-10-17

**Authors:** Hui Jiang, Quansheng Chen

**Affiliations:** 1 School of Electrical and Information Engineering, Jiangsu University, Zhenjiang 212013, China; E-Mail: h.v.jiang@ujs.edu.cn or h.v.jiang@hotmail.com; 2 School of Food and Biological Engineering, Jiangsu University, Zhenjiang 212013, China

**Keywords:** solid state fermentation, electronic nose, near infrared spectroscopy, fusion, monitoring

## Abstract

In order to assure the consistency of the final product quality, a fast and effective process monitoring is a growing need in solid state fermentation (SSF) industry. This work investigated the potential of non-invasive techniques combined with the chemometrics method, to monitor time-related changes that occur during SSF process of feed protein. Four fermentation trials conducted were monitored by an electronic nose device and a near infrared spectroscopy (NIRS) spectrometer. Firstly, principal component analysis (PCA) and independent component analysis (ICA) were respectively applied to the feature extraction and information fusion. Then, the BP_AdaBoost algorithm was used to develop the fused model for monitoring of the critical time in SSF process of feed protein. Experimental results showed that the identified results of the fusion model are much better than those of the single technique model both in the training and validation sets, and the complexity of the fusion model was also less than that of the single technique model. The overall results demonstrate that it has a high potential in online monitoring of the critical moment in SSF process by use of integrating electronic nose and NIRS techniques, and data fusion from multi-technique could significantly improve the monitoring performance of SSF process.

## 1. Introduction

As the feed protein shortage has become a global crisis and the protein demands for animal feeding increase for improving human living standards, the protein production from cellulosic resources for animal feeds are being considered worldwide [1]. Cellulose resources are abundant in nature, especially in China, but their low protein content and low digestibility prevent their use as feed for non-ruminant animals in feedlots [2]. Bioconversion of these resources by solid state fermentation (SSF) has been widely used due to its low cost, easy operation, and variety of uses [3–6]. The microorganisms produce cellulase, degrade components of the cell wall and synthesize microbial protein by SSF.

One of the main goals of modern industrial fermentation is to achieve as high consistency and reproducibility as possible. In this context, accurate monitoring of fermentation process (*i.e*., correct discriminate the critical moment in the fermentation process) is a growing need which calls for rapid tools providing in-time information in order to assure some necessary measures at all stages of the process according to a prespecified trajectories [7]. Unfortunately, there is a lack of fast, reliable, and robust measurement techniques for real-time monitoring of the critical time in the SSF process. Therefore, some rapid and non-invasive analytical tools are essentially required to monitor the fermented process by use of multivariate calibration models.

The electronic nose is an innovative device based on an array of gas sensors with different selectivities able to recognize volatile organic compounds (VOCs). The sensor array in the electronic nose system consists of some non-specific sensors, and an odor stimulus generates a characteristic fingerprint from the sensor array [8]. In recent years, numerous attempts are reported in the literature on the use of the electronic nose technique to analyze the process of SmF [9–12], and there are only several papers concerning the monitoring of the aroma evolution during SSF of tea [13–15]. However, little attention, up to the present, has been reported on the monitoring of the critical moment in SSF process of feed protein by use of electronic nose technique.

Near-infrared spectroscopy (NIRS), as a rapid and non-destructive analysis technique, represents an alternative to traditional chemical approach for measurement of various products and processes, given that absorption in the spectral regions can be related to the specific chemical components of the tested sample. In particular, the spectrum is based on electromagnetic absorption in the region from 700 to 2500 nm caused by the overtone and combination bands of the fundamental bending and stretching vibrations. The importance of NIRS in the identification of molecular structures originates from the abundant information content obtained and the possibility to assign certain absorption bands related to its functional groups such as C-C, C-H, O-H, C-O and N-H [16,17]. Recently, NIRS technique has already been employed in the field of fermentation, both in the submerged fermentation (SmF) [18–23] and in the SSF [24–27] processes. Limited references are available in the literature concerning the application of NIRS technique in monitoring the critical time of SSF process of feed protein.

Moreover, the fermentation is a very complex dynamic process due to the action of microorganisms, including a combination of chemical, biological and transport phenomena. Thus, a single detection technology can only describe one of the aspects, which is difficult to fully reflect the status of the fermentation, such as the separate electronic nose system cannot investigate molecular changes during the fermentation process, and the isolated NIRS analysis technique is not able to follow the evolution of the volatile organic compounds profile. In order to evaluate comprehensively the fermented process, some references are available in the literature concerning the application of multi-technology fusion (such as electronic nose, NIRS, *etc*.) in the field of SmF [7,28–30]. However, few studies have been reported on the process monitoring of SSF in a pilot scale by using multi-sensor data fusion technology.

The present paper reports the results of the application of electronic nose and NIRS techniques for the monitoring of the critical moment in the SSF process of feed protein. The aim is to take advantage of the two techniques, one performing a local measurement of inner chemical property of the fermentation substrate, the other one performing a global assessment of the physical properties of the fermentation substrate, and to integrate those types of measurements (local and global) to enhance the process monitoring. The specific objectives of this study were:

1. to explore the latent variables from the two sensors data by use of principal component analysis (PCA);
2. to optimize and extract the characteristic vectors in the process of the single technique (*i.e*., electronic nose or NIRS technique) model calibration;
3. to fuse the best combination of the feature vectors extracted by using independent component analysis (ICA) and to develop the optimal identification model for monitoring of the critical time in the SSF process of feed protein.

## 2. Materials and Methods

### 2.1. Fermentation Trials and Sampling

Samples were prepared at different times from four runs of SSF of protein feed trials. The rice chaff was obtained from the Zhenjiang city of Jiangsu Province of China. It was mixed with corn flour and wheat bran (rice chaff: corn flour: wheat bran was 7:2:1), and then the mixtures were ground by use of a crushing machine with 40 mesh screen. Finally, the basal substrates, which were made up of mixture of effective microorganisms (EM) bacterial liquid, water and the pretreated mixtures in the ratio of 1:200:500, were loaded in GTG-100 bioreactor (100 L) with a 40% occupation coefficient of the bioreactor volume, and cultures were incubated by anaerobic fermentation at 30 ± 2 °C for six days.

The samples were obtained by 4 runs of fermentation, and in each run, samplings were carried out at seven different time points during SSF of protein feed, from loading to the end of protein feed fermentation (0, 24, 48, 72, 96, 120 and 144 h or day 0, day 1, day 2, day 3, day 4, day 5, and day 6). At each sampling point, five semi-solid state samples taken out for signal acquisition of the near infrared spectrometer and the electronic nose system, and 35 samples were collected in every run. Thus, a total of 140 samples date could be obtained in this process.

### 2.2. Sample Set Division

In this study, all 140 fermented samples from the four runs of fermentation trial were divided into two subsets. One of the two subsets was called the training set where all samples were used to calibrate model, and the other one was called the validation set where all samples were applied to verify the robustness of the established model. The training set contained 105 samples from the first three runs of fermentation experiments, and the remaining 35 samples from the last run of fermentation trial constituted the validation set.

### 2.3. Electronic Nose Data Acquisition

The VOCs in fermented samples obtained were analyzed by use of an electronic nose system, which was designed and developed by the Agricultural Product Processing and Storage Lab of Jiangsu University. The system consists of a micro-pump with programmable sequence control, a sensor array, and a PC-based data acquisition and an automated operation by controlled software, *etc*. The sensors array is determined on the basis of their response sensitivity toward the VOCs in fermented materials, and composed of a set of eleven gas sensors from Figaro Co. Ltd., Osaka, Japan (*i.e*., universal sensors of TGS2602, TGS2610, TGS2611, TGS813, and TGS822, special sensors of TGS822TF, TGS825, TGS826, TGS880, TGS4160, and TGS5042) for odor capture in SSF process of feed protein. The sensor response is expressed as ppm.

The electronic nose experimental cycle consists of the automated sequence of internal operations for each sample contained the following three stages: (i) headspace generation; (ii) sampling; and (iii) purifying. The PC-based data acquisition and automated operation of each sampling cycle is controlled by use of specially designed software. The software has the features like programmable sequence control, data logging, alarm annunciation, *etc*. The software has been developed in Delphi 7 of Borland Software Corporation. The experimental conditions of the electronic nose system for monitoring of the process of SSF of feed protein are given as follows:

- Amount of each fermented sample = 6 g,
- Temperature = 22 ± 1 °C,
- Headspace generation time = 120 s,
- Sampling time = 120 s,
- Purging time = 180 s,
- Airflow rate = 3 mL/s.

### 2.4. Near Infrared Spectra Measurement

The NIR spectra were collected with the reflectance mode (Log 1/R) using an Antaris^TM^ II FT-NIR spectrometer (Thermo Scientific Co., Waltham, MA, USA) equipped with an integrating sphere. Each spectrum was the average of 16 scanning spectra. The NIR measurements were performed within the region 10,000 to 4000 cm^−1^, and the data were acquired with a spectral resolution of 3.856 cm^−1^, which resulted in 1557 variables. When spectra collecting, about 6 g of semi-solid state fermented sample without pretreatment as a sample, which was put in a standard sample cup particularly designed by Thermo Scientific Co. Each sample was collected three times at different positions, and the three spectra were averaged to provide a mean spectrum for each sample was used in the next analysis. In NIR spectra collection, near infrared spectrometer was sensitive to the change of outer environment condition such as temperature and humidity. Therefore, the temperature was kept around 25 °C and at a steady humidity level in the laboratory.

### 2.5. Data Processing Methods

#### 2.5.1. Principal Component Analysis

Principal component analysis (PCA) is a linear pattern recognition technique for describing the unique variances (*i.e*., principal components, PCs) via linear combinations of the original variables, and these PCs are orthogonal. It can effectively extract useful information and eliminate redundant ones, and similar samples will be located close to each other after transformation. Thus, the graphical output can be used for exploring the difference between groups and comparing this difference to the distribution pattern within one group [31].

#### 2.5.2. Independent Component Analysis

Independent component analysis (ICA) [32] is a multivariate data analysis method that, given a linear mixture of statistical independent sources, recovers these components by producing an unmixing matrix. Stemming from a more general problem called blind source separation (BSS), ICA has become increasingly popular in recent years. In the basic form of ICA, let us denote by *x* = [*x*_1_,*x*_2_,…,*s_m_*]*^T^* an *m*-dimension observation vector, and by *s* = [*s*_1_,*s*_2_,…,*s_m_*]*^T^* an *n*-dimension unknown independent components (ICs). The ICs are mutually statistically independent. Then the linear relationship is given by

(1) x=As

where matrix *A_m_*_×_*_n_* is an unknown matrix of full rank, called the mixing matrix. The basic goal is to find an *n* × *m* separating matrix *W* without knowing the mixing matrix *A*, that is

(2) $$y=W^{T}x$$

such that *y* = [*y*_1_,*y*_2_,…,*y_n_*]*^T^* is an estimate of *s* in the sense that each component of *s* may appear in any component of *y* with a scalar factor. Here it is assumed that the mixing matrix is square (*n* = *m*). If the number of ICs vectors is greater than the dimensionality of the observed vectors, *n* > *m*, the task is overcomplete but is still solvable with the pseudo inverse.

#### 2.5.3. BP_AdaBoost Algorithm

In this study, BP_AdaBoost algorithm was employed to build the identification model for the monitoring of the process of SSF. Generally, the defect of the classical BP neural network is easy to fall into local minimum and cannot get global optimum. The selection of hidden layer nodes in the network lacks of instruction in theory, and it is usually determined on the basis of experience. Thus, the network often has a great redundancy, also potentially increase the learning time. AdaBoost is a simple and effective iterative learning algorithm, the idea of which is attempt to construction a classifier of strong classification ability through a combination of a series of classifier of weak classification ability. In order to solve the defects of the BP neural network such as slow converging speed, a model combining BP neural network with AdaBoost algorithm named as BP_AdaBoost [33] was presented as the following steps:

Step1: Data selection and network initialization. Select *m* group training samples from samples space; initialize the distribution weight *D_t_*(*i*) = 1/*m* of training samples. Establish the structure of BP neural network by the dimensions of input and output samples, and initialize the weights and thresholds of BP neural network.
Step2: The prediction of weak predictor. When training the *t* weak predictor, training BP neural network with the training samples *g*(*t*) and forecasting the output. Obtain the summation of prediction error *e_t_* of the training samples, the expression is as follows:
  (3) $$\begin{array}{cc} e_{t}=\underset{i}{\text{∑}}D_{i}(i) & i=1,2,\ldots ,m \end{array}$$
  (4) $$\begin{array}{cc} D_{t+1}(i)=\{\begin{array}{c} kD_{t}(i)\\ D_{t}(i) \end{array} & \begin{array}{c} if\begin{array}{c} \left|err\right|>0.2 \end{array}\\ else \end{array} \end{array}$$
  where *k* is the adjustment factor of the distribution weight, and *k* = 1.1 generally; err = *y_i_* − *ŷ_i_* is the predicted error of the training samples, *y_i_* and *ŷ_i_* is the expected values and predicted values of the training samples *g*(*t*).
Step3: Calculate the weight of the prediction sequence. Calculate the weight *a_t_* by *e_t_* the summation of forecasting error of training samples *g*(*t*).
  (5) $$a_{t}=\frac{1}{2}Ln(\frac{1-e_{t}}{e_{t}})$$
Step4: Test data and adjustment weight. Adjust the weight of the next training samples by the weight of the prediction sequence *a_t_*, its mathematical expression is
  (6) $$\begin{array}{cc} D_{t+1}(i)=\frac{D_{t}(i)}{B_{t}}*\exp[-a_{t}y_{i}g_{t}(x_{i})] & i=1,2,\ldots ,m \end{array}$$
  where *B_t_* is the normalization factor, it make the summation of distribution weights is 1 under the same weight ratio.
Step5: Strong prediction function. Normalized gained weight of *T* weak predictors function *f*(*g_t_*,*a_t_*). Then the predictions of strong prediction function *h*(*x*) is as follows:
  (7) $$h(x)=sign[\overset{T}{\underset{t=1}{\text{∑}}}a_{t}\cdot f(g_{t},a_{t})]$$

In BP_AdaBoost model calibration, the model was optimized by a leave-one-out cross-validation, and the optimal number of input variables was determined according to the first local highest identification rate. In this study, it was implemented as follows: (1) the spectrum of one sample was left-out in the training set, and a model was built with the remaining samples in the training set; (2) the left-out sample was identified by this model, and the procedure was repeated by eliminating each sample in the training set; (3) the identification rate was then calculated according to the identification result and the real category of each sample in the training set. In addition, in order to establish a best identification model, a dummy numeral was assigned to each of the samples. The prediction value of the BP_AdaBoost model is a real number, not a dummy integer. In order to determine which class a sample belongs to, a cutoff value needs to be set. In this study, the cutoff value was set as 0.5.

### 2.6. Software

NIR spectra of the fermented samples were acquired and stored by software (Antaris^TM^ II System, Thermo Scientific Co., Waltham, MA, USA). Software of electronic nose data acquisition was compiled by us based on Delphi 7 (Borland, Scotts Valley, CA, USA). All algorithms were implemented in PASW Statistics 18 (IBM, New York, NY, USA) and Matlab R2010a (Mathworks, Natick, MA, USA) under Windows 7 in data processing.

## 3. Results and Discussion

### 3.1. Results of PCA

Before performing PCA calculation, the raw NIR spectral data were preprocessed by standard normal variate (SNV). And for the electronic nose signals, the maximum value of the response of each gas sensor was extracted as the initial latent variables of the raw electronic nose signals. In this study, there are some overlapped information existing in the NIRS and electronic nose data, and these collinear variables might bring severe difficulty to the research. This problem can be solved by use of PCA that is the data reconstruction and dimensional reduction method. In addition, a score plot was obtained by use of the top three PCs extracted from the normalized characteristic values, to visualize the cluster tendency of all the fermented samples.

**Figure 1.**
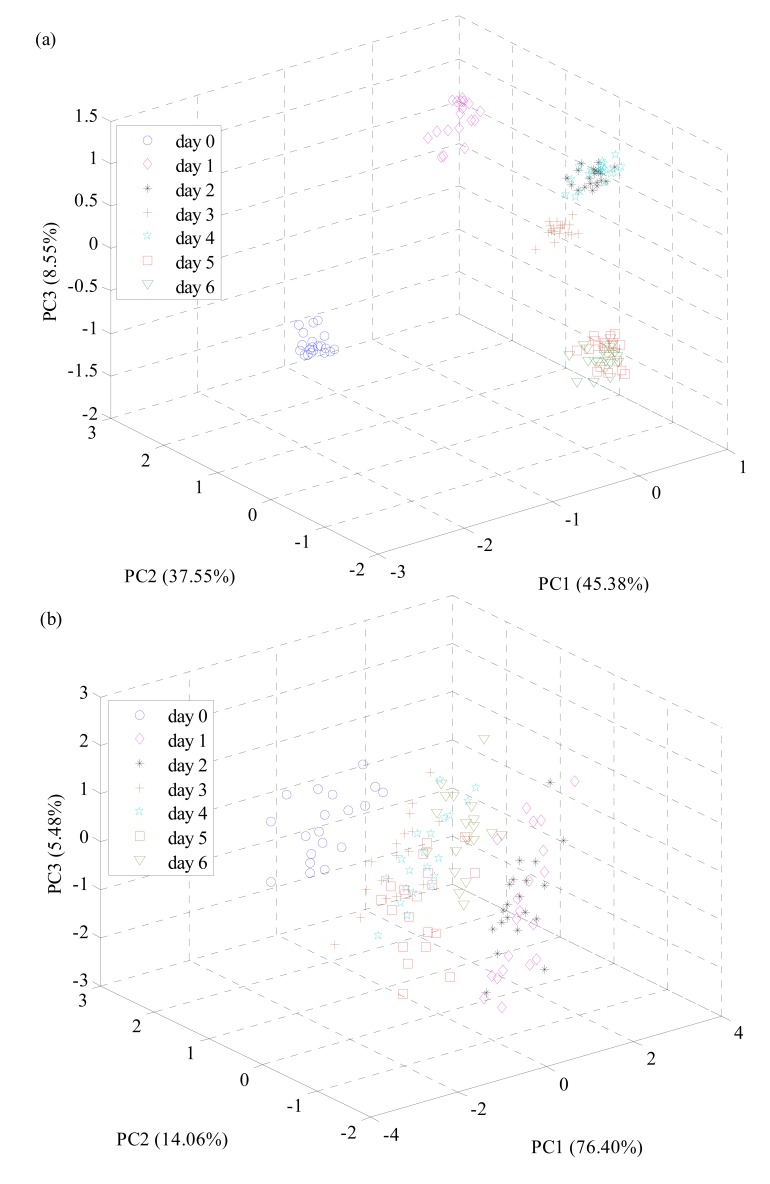
Score cluster plot with the top three principal components (PCs) from all samples. Score cluster obtained from (**a**) electronic nose data and (**b**) near infrared spectroscopy (NIRS) data.

Figure 1a shows a three-dimensional scatter plot represented by the top three PCs (*i.e.*, PC1, PC2, and PC3) issued from PCA based on the electronic nose signals. PC1 can explain 45.38% variances, PC2 can explain 37.55% variances, and PC3 can explain 8.55% variances. The cumulative variances contribution rate of PC1, PC2 and PC3 is 91.48%. Investigated from Figure 1a, seven sample groups appeared in cluster trend along three principal component axes, confirming the presence of several different clusters just associated with their fermented degree. The samples from “day 0”, “day 1”, and “day 3” could be separated directly by PCA. Nevertheless, the separation of the samples from “day 2” and “day 4” was not clear, especially, more overlap can be observed from the samples of “day 5” and “day 6”. It shows that the samples of “day 5” is similar to the samples of “day 6” in their internal ingredients, and can be inferred that the fermentation process has already completed basically when the fermentation to the fifth day.

Figure 1b shows the score plot constructed by PC1, PC2 and PC3 derived from PCA based on the spectral data. PC1 can explain 76.40% of the variance, PC2 can explain 14.06% of the variance, and PC3 can explain 5.48% of the variance. The total accumulative contribution rate of variance from PC1, PC2, and PC3 is 95.94%. As seen in Figure 1b, the separation between samples of different fermented state was not so satisfied, and samples of different groups were overlapped. Besides, samples from one group were also highly scattered and no clear cluster. Only samples from “day 0” were generally separated from samples of other groups. The results indicated that chemical differences between these samples might be small.

### 3.2. Results of Single Technique Model

The geometrical exploration of 3D plot by PCA can give the cluster trend of the all fermented samples and cannot be used as an identification tool. Therefore, the pattern recognition methods could be used to identify the samples of different fermented degree. Before building identification model, the principal component factors, as the inputs of model, were extracted by PCA, and the number of PCs was optimized in model calibration. In this study, the BP_AdaBoost algorithm was employed to calibrate the monitoring model based on different techniques.

In model calibration, the BP neural network with 3 layers construction was used to calibrate the identification model. The parameters of the network were optimized by a cross-validation as follows: the number of neurons in the hidden layer was set to 10, the learning rate factor and momentum factor were set to 0.1, the initial weight was set to 0.3, and the scale function was set as “Sigmoid” function; the permitted training error was set to 0.01; the maximal times of training were set to 50. For AdaBoost algorithm, the number of iterations was set to 10 on the basis of experience. It is crucial to choose the appropriate number of PCs in building BP_AdaBoost model. So the number of PCs should be optimized by the cross-validation in model calibration, and the optimal number of PCs was finally determined by the consideration of the identification rates of the models in the training and validation sets.

**Figure 2.**
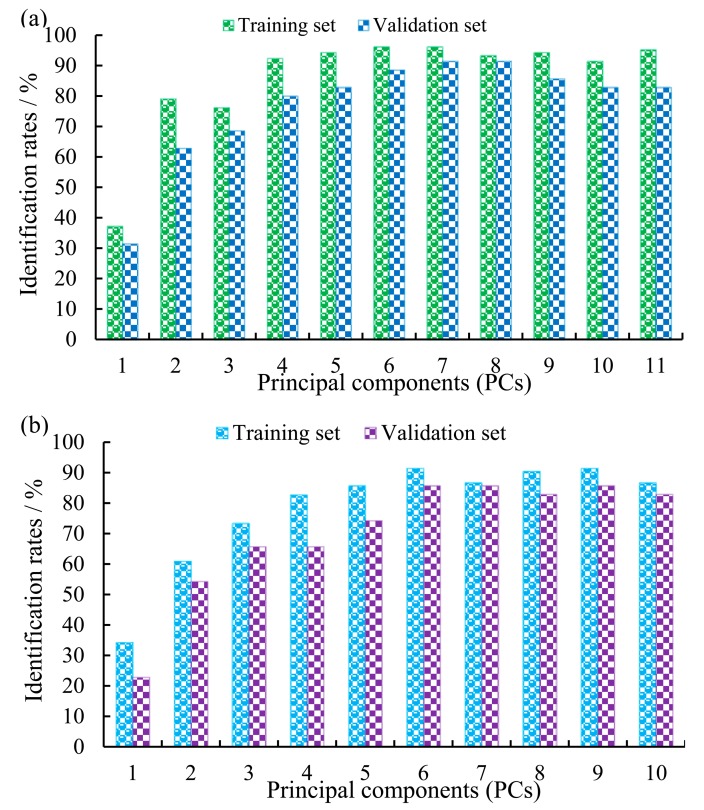
Identification rates of the identified model with a different number of PCs in training and validation sets. Results obtained from (**a**) electronic nose data and (**b**) NIRS data.

Figure 2a shows the identification rates of electronic nose technique model with different PCs in the training and validation sets. It can be found from Figure 2a, the optimal model was achieved when PCs was equal to seven. The identification rates of the model was 96.19% in the training set and 91.43% in the validation set, respectively. Therefore, the best number of PCs of the model based on the electronic nose technique was seven.

Figure 2b shows the identification rates of NIRS technique model with different PCs in the training and validation sets. Investigated from Figure 2b, the best model was obtained when six PCs were included. The identification rates of the model was 91.43% in the training set and 85.71% in the validation set, respectively. Thus, the optimal number of PCs of the model based on NIRS technique was six.

### 3.3. Results of the Two-Technology Fusion Model

**Figure 3.**
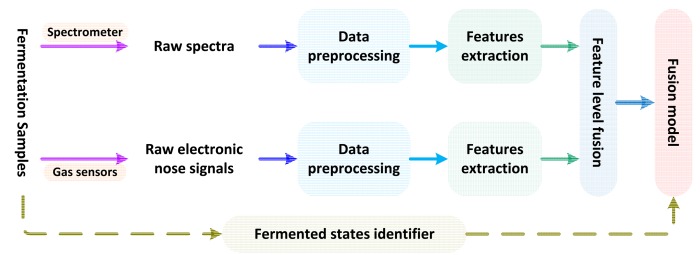
The structure of the intermediate-level fusion for monitoring of SSF of feed protein.

In this study, in order to make full use of the different sensors data, information fusion technology will be used to improve the reliability of the monitoring of the critical moment in SSF process of feed protein. In general, the information fusion can be divided into three levels, *i.e*., low-level fusion, intermediate-level fusion, and high-level fusion [34]. The low-level fusion is direct integration of the raw data of the various sensors also called original data fusion, and it has the requirement of consubstantial sensors, high cost, time-consuming and bad real-time. The high-level fusion, namely decision-making fusion, need a knowledge base of the target, and its drawback is that has a huge cost on data preprocessing and easy to cause tremendous information losses. Compared with the low-level fusion and the high-level fusion, the intermediate-level fusion, also known as feature level fusion, is the integration of the feature variables of each sensor, which can keep enough raw information, and also can eliminate redundant information. Therefore, in this study, the combination of feature vectors from electronic nose and NIRS data will be fused by ICA algorithm at feature-based level (*i.e*., intermediate-level fusion) for further analysis. Figure 3 presents the structure of the intermediate-level fusion for the process monitoring of SSF of feed protein.

According to mentioned above, the optimal BP_AdaBoost model was obtained based on the NIRS technique when the number of PCs was equal to six, and the best BP_AdaBoost model was achieved based on the electronic nose technique when seven PCs were used. In other words, the best combination for PCs (7, 6) was obtained based on the electronic nose and NIRS techniques model. Considering the characteristic variables from different techniques might still exist some correlative and redundant information. In this study, ICA was employed to fuse the feature combination from the single technique. Similarly, the number of ICs was also optimized by cross-validation in model calibration, and determined by leveraging the classification rates of the BP_AdaBoost model in the training and validation sets to get the best fused model of two-technique.

**Figure 4.**
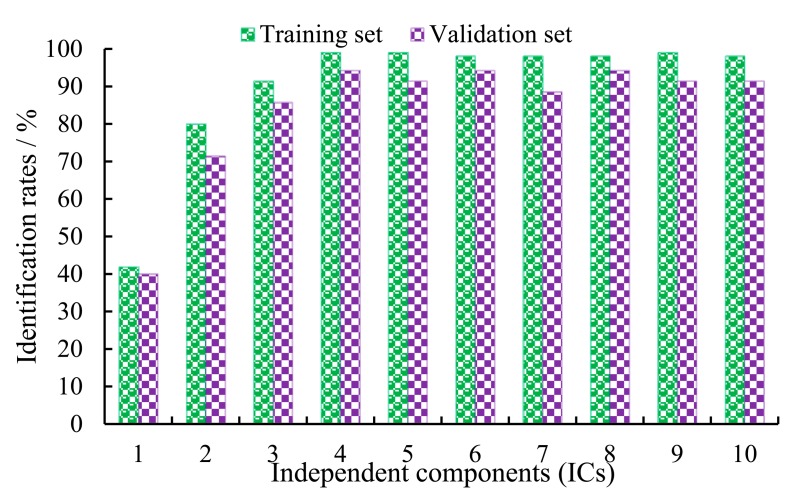
Identification rates of the fused model with a different number of ICs in training and validation sets.

Figure 4 shows the identification rates of the BP_AdaBoost model with different ICs in the training set and validation set. The optimal BP_AdaBoost model for monitoring of the different fermented status was obtained when the number of ICs was equal to four. The identification rate was 99.05% in the training set, while predictive identification rate in the validation set was 94.29%.

Table 1 shows the detailed identification results from the best BP_AdaBoost model. On detailed identification results, the figure of diagonal line mean the number of samples which was identified correctly, and the rest on behalf of the number of samples of misclassification. As can be seen from Table 1, some misclassifications usually occur between “day 5” samples and “day 6” samples both in the training set and in the validation set. As for the reasons, we can infer that there are very small differences existing in “day 5” samples and “day 6” samples so that some samples cannot be effectively differentiated by the fusion model based on the NIRS and electronic nose techniques. Usually, along with the fermentation, the whole process can be divided into three stages, *i.e.*, lag phase, exponential phase, and stationary phase. These changes of fermented substrate tissue are very slow or even stagnant because the fermentation has entered into stationary phase when the fermentation to the fifth day. Therefore, it is reasonable that the misclassifications only occur between “day 5” samples and “day 6” samples.

### 3.4. Discussion of Identification Results

In order to further highlight the main idea of this work, the results of the fusion model of multi-technique were compared with those of the single model in this study. The best results from the different models are presented in Table 2. Investigated from Table 2, comparing the models of different techniques, the models based on data fusion of NIRS and electronic nose techniques are superior to those of two single technique. We could give detailed discussions for the results obtained as follows:

As seen from the aspect of technique, vibration and combination overtones of the fundamental O-H, C-H, S-H and N-H bonds are the main recordable phenomena in the NIR region. The importance of NIRS in the identification of molecular structures originates from the abundant information content obtained and the possibility to assign certain absorption bands related to its functional groups including these hydrogenous bonds. During SSF, the process is often accompanied with the changes from internal attributes such as chemical components and physical properties, which results in great changes in the structure of these specific functional groups that can be related to some certain absorption bands in the NIR region. Thus, the differences between different samples from different fermented degrees can be reasonably existed in near infrared spectra, and provide the possibilities of classifying the fermented samples with different states by NIRS technique. At the same time, along with the fermentation, the differences of the emitted smells of the fermented materials can also be responded by electronic nose technique. Actually, the processes of SSF of feed protein are very complex due to involving growth of microorganism, thus the single detection technology can only describe one of the aspects, which is difficult to fully reflect the state of the fermented material. And the multi-technology fusion based on different sensors data can acquire more information than single sensor data from the fermented samples and more fully reflect the status of fermented processes. Therefore, it can more fully explain the fermented degree of SSF of feed protein, and thus get better identification result for monitoring of the process.

## 4. Conclusions

The results of this study demonstrate a good ability of electronic nose and NIRS techniques to follow the fermentation process giving crucial information about the state of the SSF process of feed protein. Compared with the single technology model, the modeling of electronic nose and NIRS data shows that the combination of these non-destructive tools could significantly improve identification performance of the process state of SSF of feed protein. Therefore, it can be also concluded that these devices can be regarded as valid and simple instruments, able to provide real-time signals during SSF in order to monitor the development of the process from different perspectives. Although in this work the measurements were carried out via the offline mode, Invasive sensors or probes could be performed in future works to allow online monitoring of the process, giving real-time information which relate to the development of the process and to the quality of the final product.

## Figures and Tables

**Table 1.** Detail identification results of the best fusion model in the training and validation sets.

**Sample Set**	**Sample Number**	**Fermentation State**	**Detailed Identification Results**							**Identification Rates/%**

			**Day 0**	**Day 1**	**Day 2**	**Day 3**	**Day 4**	**Day 5**	**Day 6**	
Training set	15	day 0	15	0	0	0	0	0	0	100
	15	day 1	0	15	0	0	0	0	0	100
	15	day 2	0	0	15	0	0	0	0	100
	15	day 3	0	0	0	15	0	0	0	100
	15	day 4	0	0	0	0	15	0	0	100
	15	day 5	0	0	0	0	0	15	0	100
	15	day 6	0	0	0	0	0	1	14	93.33

Validation set	5	day 0	5	0	0	0	0	0	0	100
	5	day 1	0	5	0	0	0	0	0	100
	5	day 2	0	0	5	0	0	0	0	100
	5	day 3	0	0	0	5	0	0	0	100
	5	day 4	0	0	0	0	5	0	0	100
	5	day 5	0	0	0	0	0	5	0	100
	5	day 6	0	0	0	0	0	2	3	60

**Table 2.** Results and comparison of the best BP_AdaBoost models based on different techniques.

**Technical Models**	**Identification Results**				

	**Latent Vectors**	**Training Set**		**Validation Set**	

		**Ratio**	**%**	**Ratio**	**%**
NIRS	6	96/105	91.43	30/35	85.71
Electronic nose	7	101/105	96.19	32/35	91.43
Fusion	4	104/105	99.05	33/35	94.29
